# Prediction of Intact N-Glycopeptide Retention Time Windows in Hydrophilic Interaction Liquid Chromatography

**DOI:** 10.3390/molecules27123723

**Published:** 2022-06-09

**Authors:** Petr Kozlik, Katarina Molnarova, Tomas Jecmen, Tomas Krizek, Zuzana Bosakova

**Affiliations:** 1 Department of Analytical Chemistry, Faculty of Science, Charles University, Hlavova 8, 128 43 Prague, Czech Republic; katarina.molnarova@natur.cuni.cz (K.M.); krizek@natur.cuni.cz (T.K.); bosakova@natur.cuni.cz (Z.B.); 2 Department of Biochemistry, Faculty of Science, Charles University, Hlavova 8, 128 43 Prague, Czech Republic; tomas.jecmen@natur.cuni.cz

**Keywords:** glycoproteomics, glycopeptides, glycopeptide separation, haptoglobin, hemopexin, hydrophilic interaction liquid chromatography, retention time prediction, sex hormone-binding globulin

## Abstract

Analysis of protein glycosylation is challenging due to micro- and macro-heterogeneity of the attached glycans. Hydrophilic interaction liquid chromatography (HILIC) is a mode of choice for separation of intact glycopeptides, which are inadequately resolved by reversed phase chromatography. In this work, we propose an easy-to-use model to predict retention time windows of glycopeptides in HILIC. We constructed this model based on the parameters derived from chromatographic separation of six differently glycosylated peptides obtained from tryptic digests of three plasma proteins: haptoglobin, hemopexin, and sex hormone-binding globulin. We calculated relative retention times of different glycoforms attached to the same peptide to the bi-antennary form and showed that the character of the peptide moiety did not significantly change the relative retention time differences between the glycoforms. To challenge the model, we assessed chromatographic behavior of fetuin glycopeptides experimentally, and their retention times all fell within the calculated retention time windows, which suggests that the retention time window prediction model in HILIC is sufficiently accurate. Relative retention time windows provide complementary information to mass spectrometric data, and we consider them useful for reliable determination of protein glycosylation in a site-specific manner.

## 1. Introduction

Glycoproteomic analysis is highly challenging due to the huge diversity of glycan structures attached to proteins, their low abundance, which is linked to their site-specific heterogeneity, and poor ionization efficiency of glycans and glycopeptides in mass spectrometry [1,2,3,4]. A combination of liquid chromatography with tandem mass spectrometry, followed by spectral matching of MS/MS fragment masses to an in silico database of theoretical combinations of a glycan, and a peptide is the standard technique in glycopeptide analysis [4]. Many glycoproteomic search engines have been developed in the last decade that allow automated characterization of intact glycopeptides [5,6,7,8]. The approaches based solely on MS identification suffer from a high rate of ambiguous annotations [8] and require a good degree of manual curation [9] as they tackle with difficulties, such as unavailability of high-quality information-rich MS/MS spectra, a presence of unexpected glycans or peptide modifications, and the occurrence of glycan and amino acid combinations of equivalent mass [3,5]. Liquid chromatography, an integral part of the vast majority of structural elucidation workflows in proteomics [10,11,12], provides additional information that is orthogonal to mass spectrometric data and can, therefore, be included in the glycopeptide search algorithms. In recent years, several studies have focused on improving the accuracy of glycopeptide identification by also considering their retention time in reversed-phase (RP) chromatography [5,10,13,14,15,16]. Although RP is the most used chromatographic mode for glycopeptide analysis, it provides low selectivity for different glycoforms attached to the same peptide backbone [14]. HILIC is an alternative chromatographic mode for glycopeptide separation that provides higher selectivity of different glycoforms compared to RP mode due to the significant contribution of glycan structure on the retention [17,18,19,20,21,22]. To date, there is a limited number of publications that explore the potential of including glycopeptide retention times in HILIC to improve their identification [21,23].

In this work, we selected three clinically relevant plasma glycoproteins: haptoglobin, hemopexin, and sex hormone-binding globulin [24,25,26]. We investigated retention behavior of their tryptic glycopeptides in HILIC in a nanoscale setting. We used HALO^®^ penta-HILIC stationary phase that showed a high separation efficiency for glycopeptide in our previous study [20]. Based on the chromatographic data, we derived an easy-to-use mathematical model to predict the retention time windows of glycopeptides, which are relative to a retention time of a glycopeptide identified with high fidelity, in HILIC. We then showed, in the case of fetuin glycopeptides, that the prediction based on the proposed model was consistent with the measurement. The presented concept of relative retention time windows thus shows potential to reduce glycopeptide false discovery rate, one of the critical pitfalls of current glycoproteomic search engines, when included in the post-search filtering of the glycopeptide assignments.

## 2. Results and Discussion

In this study, we compared the retention times of different glycoforms attached to different peptide backbones in HILIC mode. For this purpose, we prepared tryptic digests of three human serum glycoproteins (haptoglobin, hemopexin, and sex hormone-binding globulin), each providing two peptides with a single N-glycosylation site. We used retention times obtained for the glycoforms of these peptides, which differed in monosaccharide unit composition, to determine relative retention windows of the respective glycoforms. All studied glycopeptides are outlined in Table 1.

### 2.1. Relative Retention Time

**Figure 1:**
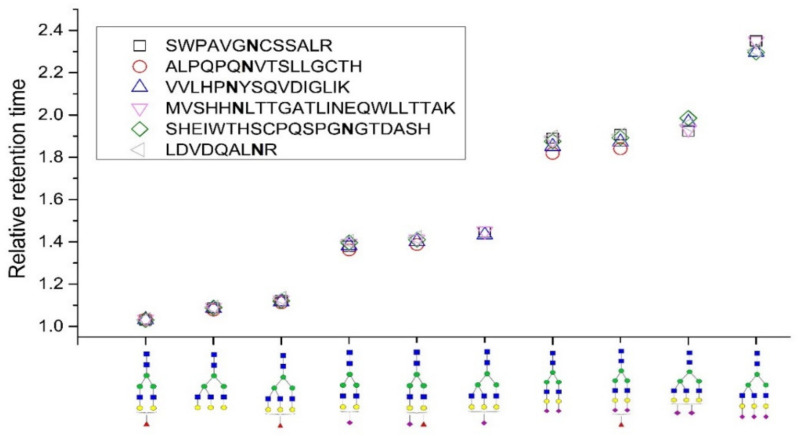
Relative retention times (*t*r_x glycoform_/*t*r_A2G2 glycoform_) of individual glycoforms attached to different peptides.

All samples were injected four times, and the retention time of the analytes was highly reproducible (RSD 0.6%). We observed extensive glycopeptide interaction with the used stationary phase in HILIC, and differences in glycan structure were manifested as intense retention shifts in this chromatographic mode. The SRM chromatograms of all the studied glycopeptides of hemopexin, haptoglobin, and sex-hormone binding globulin are shown in Figures S1–S3. Glycoform A2G2 always had the shortest retention time from all the glycoforms attached to the same peptide backbone, and the retention of the other glycoforms increased depending on the number and type of monosaccharide units extending the glycan. As we show here and as described in our previous studies [17,20], the effect was notable for neutral monosaccharides and more profound for sialic acids. Individual glycoforms attached to peptides differing in amino acid sequence were eluted at different retention times; for example, the glycoform A2G2 of the LDVDQALNR peptide was eluted in 35.2 min while the same glycoform of the SWPAVGNCSSALR peptide was eluted in 28.9 min. However, the character of the peptide backbone did not significantly alter the relative retention time (RRT) of the individual glycoforms, which was calculated as RRT = *t*r_(glycoform x)_/*t*r_(glycoform A2G2)_. It supports our idea to use that retention behavior for prediction of glycopeptide retention time windows. We document this in Figure 1, where we show the retention time of individual glycoforms attached to different peptides relative to the respective A2G2 glycoform. Here, only highly sialylated glycoforms exhibit slightly higher variability in RRTs, most likely due to high retention shift from the A2G2 glycoform.

Using the same HILIC stationary phase but a different gradient program with a nonidentical mobile-phase composition, chromatographic behavior consistent with our expectation was observed in the separation of fetuin and IgG glycopeptides [19], which suggests that this observation can be generalized for a broader range of experimental conditions and glycopeptides with various peptide backbones. However, in contrast to our approach, Badgett et al. [21] was expressing the glycopeptide retention relatively to the external reference, which used retention coefficients of a dextran ladder for calibration. Although we consider this approach to be well applicable for the purpose of bare glycan chromatographic behavior assessment, we see it to be currently applicable only to a limited extent in prediction of glycopeptide retention. This is discussed in more details below.

Distinct glycopeptide glycoforms are poorly separated in RP mode compared to HILIC. An additional neutral monosaccharide unit causes the glycopeptide to elute only slightly earlier, whereas an extra sialic acid prolongs glycopeptide retention relatively to the respective counterpart [5,18]. As a consequence, glycopeptides with the same peptide backbone elute in a narrow retention window, which can be determined when at least one of the glycoforms in the cluster is reliably identified, similar to the approach we propose here. These narrow retention time windows of glycopeptides in RP mode were implemented as a search parameter, resulting in improved identification accuracy of AXL receptor tyrosine kinase glycopeptides treated with exoglycosidase [5] or revealing the site-specific glycome of complex mixtures without requiring tandem mass data [27]. The RP mode can principally confirm that the unknown glycopeptide has the same peptide backbone as the other glycopeptides in the cluster, while the HILIC mode also resolves isobaric glycopeptide isomers and provides additional information about the glycopeptide retention time shift relative to the other glycoforms in the cluster, which in our opinion makes this mode superior to RP in terms of glycopeptide assignment validation based on obtained chromatographic parameters.

### 2.2. Prediction of Retention Time Windows

Absolute retention times of glycopeptides in RP can be predicted with high accuracy [13], as they are primarily a function of the peptide–amino acid sequence and depend only less significantly on the number of neutral monosaccharide units and sialic acids, whose effect on retention has already been discussed above. In contrast, prediction of absolute retention times of glycopeptides in HILIC is an intricate task and can easily lead to a false-negative identification. Both glycan and peptide moiety can affect retention substantially, as illustrated in data presented here (Figures S1–S3) and elsewhere [17,18,19,21]. The extent to which each moiety contributes is highly dependent on the HILIC stationary phase used and the chromatographic conditions applied, which we showed in our previous studies [20,22]. For these reasons, we consider prediction of retention windows—narrow intervals in which individual glycopeptides are eluted—relative to a retention time of a glycopeptide identified with high fidelity, which is a more convenient method for determination of parameters orthogonal to MS spectral data for glycopeptide search engines.

The model predicting relative retention time windows was constructed as described in the Materials and Methods section. The calculated median and lower and upper limits of the retention time windows are shown in Table 2.

**Figure 2:**
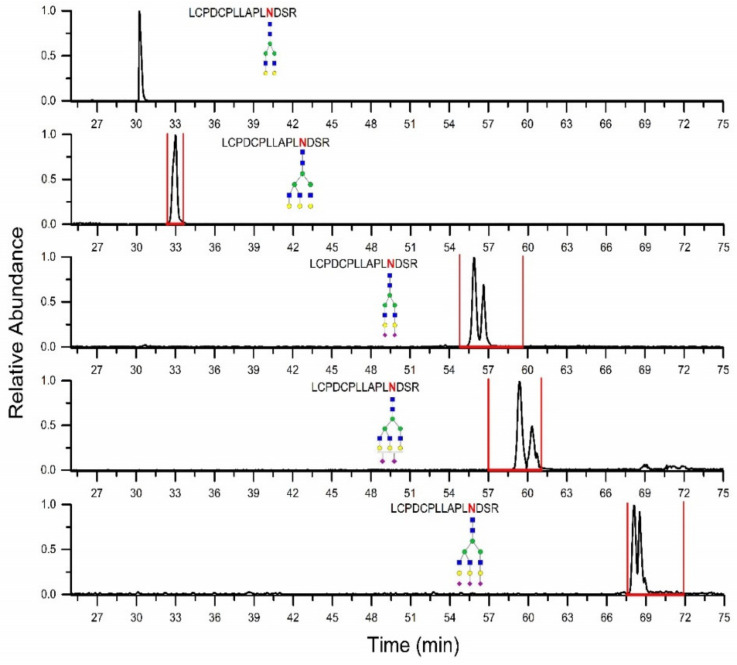
SRM chromatograms of selected fetuin LCPDCPLLAPLNDSR peptide glycoforms with retention time windows (marked by red lines) predicted according to the presented model.

To demonstrate how well the prediction of retention time windows performs, we present the results for glycoforms of LCPDCPLLAPLNDSR fetuin peptide. First, the A2G2 glycoform of the peptide was identified in LC-MS/MS data, and its retention time was used to calculate the retention time windows for the other glycoforms. The SRM chromatograms of identified glycopeptides separated on HILIC, and the retention time windows predicted based on our model are shown in Figure 2, which shows that all identified glycoforms are eluted within the predicted retention time windows. Multiple peaks for sialylated glycoforms most likely correspond to α2,3- or α2,6-linked sialic acid, as described by Huang et al. [19]. Additionally, Table 3 also shows experimentally determined retention times of the other identified fetuin glycopeptides, which all fall within the predicted retention time windows. The results presented here suggest that the glycopeptide retention prediction model in HILIC is sufficiently accurate.

As mentioned above, a conceptually similar model predicting the elution time windows of selected glycoforms of a single peptide assessed relative to the retention time of a glycoform of the same peptide identified solely based on spectral matching in RP was successfully used to increase the number of identified N-glycopeptides and decrease the false discovery rate of the analysis [5,27]. Badgett et al. showed the model for predicting the HILIC retention times of IgG glycopeptides on penta-HILIC stationary phase [21]. Their model provided accurate results for IgG and predicted glycopeptide isomeric separation. However, a major shortcoming of this model in our opinion is that it has been insufficiently validated using too few glycopeptides of similar length and minimally differing amino acid composition, which raises the question of how accurate this model would be in general. To construct this model, chromatographic behavior was, in absolute terms, first determined separately for bare glycans [19] and bare peptides [28], and then combined, which the authors proposed to correspond to the chromatographic behavior of glycopeptides. Huang et al. demonstrated that the glycopeptides and the glycans released from them are separated similarly; only a shift in retention as a consequence of the peptide moiety loss is observed [19]. The observation is also in agreement with our findings that the character of the peptide backbone does not significantly alter the relative retention times of the individual glycoforms. Badgett et al. showed that retention time of bare peptides in HILIC is strongly affected by their amino acid composition, its length, and location of amino acid residues within the peptide [28]. Moreover, two site-specific corrections for hydrophobic residues at the N-terminus and hydrophobic residues one spot over from the N-terminus were provided. It suggests that the retention mechanism of peptides in HILIC is complex, which may complicate their absolute retention time prediction. We applied the models to predict the retention of our set of glycopeptides, and setting aside the exact retention time values, which could be inaccurate due to nonidentical experimental conditions during the chromatography, the model was unable to determine the order in which the A2G2 glycoforms were eluted. We also tested another peptide retention prediction model proposed by Gilar et al., which optimized a bare silica, a bridge-ethyl hybrid silica, or an amide stationary phase [29] and ended with the same unsatisfactory result. The differences in the models were not only in retention coefficients determined for individual amino acids but also in other parameters included into the calculation. This reflects the dismal state of understanding of the mechanism by which structurally complex molecules, such as glycopeptides, are retained in HILIC. As the attempts to create an accurate general absolute retention time prediction model based solely on calculation continue to fail, we believe that the concept using relative retention windows requiring reliable identification of one glycopeptide within the cluster by spectral matching to confirm the other glycoforms of the same peptide can be used for post-search filtering of annotated results. We also believe that treating isobaric isomers as one glycoform reduces the probability of false-negatives.

Although this proof-of-concept study was performed only on a limited number of glycopeptides, we consider the proposed model for prediction of retention time windows of glycopeptides in HILIC compelling and the result conclusive. However, the development of a more elaborate glycopeptide retention prediction model would require larger datasets to cover broad structural diversity of glycopeptides. Additionally, more detailed studies are needed to better understand the impact of chromatographic conditions (especially gradient slope) on the relative retention time of glycopeptides in HILIC. The gradient slope significantly different from ours could affect RRT mainly for sialylated glycopeptides, as they eluate substantially later than the A2G2 glycoform. For this reason, if a specific application in HILIC requires major gradient slope modification, we recommend analyzing the model set of glycopeptides to adjust the retention time windows.

## 3. Materials and Methods

### 3.1. Chemicals

Acetonitrile (LC-MS grade), formic acid (LC-MS grade), iodoacetamid (purity ≥ 99%), dithiotreitol (purity ≥ 99%), sex hormone-binding globulin from human serum, bovine fetuin, and SOLu-Trypsin were purchased from Sigma-Aldrich (St. Louis, MO, USA). Hemopexin and haptoglobin standards from human plasma were purchased from Athens Research and Technology, Inc. (Athens, GA, USA). Water (LC-MS grade), ammonium hydrogen carbonate (LC-MS grade), and acetic acid (LC-MS grade) were supplied by Merck (Darmstadt, Germany). α2–3,6,8,9 neuraminidase, and GlycoBuffer 1 were purchased from New England BioLabs (Ipswich, MA, USA).

### 3.2. Sample Processing

The glycopeptide standards of hemopexin, haptoglobin, and sex hormone-binding globulin were proteolytically digested by trypsin as described earlier [14]. Briefly, 10 µL (100 µg) of each standard was diluted in 190 µL of 50 mM ammonium bicarbonate. Proteins were reduced by 5 mM dithiothreitol for 60 min at 60 °C and alkylated by 15 mM iodoacetamide for 20 min at room temperature in the dark. Residual iodoacetamide was reduced by 5 mM DTT for 30 min at room temperature. Trypsin was added at an enzyme:protein ratio of 1:25 *w*/*w* and digested at 37 °C overnight. Next, the samples were desalted using solid-phase extraction (SPE) on a Sep-Pak Vac C18 cartridge (Waters, Milford, MA, USA) according to the previously described desalting procedure [20] and eluted in 0.5 mL of 70% acetonitrile with 2% acetic acid. A 50 µL (10 µg) of the desalted standard digest was subjected to desialylation by adding 14 µL of GlycoBuffer 1 and 14 µL of neuraminidase overnight at 37 °C. The desialylated sample was desalted, as mentioned in desalting procedure [18], and eluted in 0.3 mL 70% acetonitrile with 2% acetic acid. Then, 50 µL of the glycoprotein tryptic digest was mixed with 200 µL of the desialylated sample. The mixture was evaporated and reconstituted in 65% acetonitrile with 0.1% formic acid, analyzing 1 µL by HILIC-MS/MS. Sialylated and desialylated fetuin glycopeptide standards were prepared analogously.

### 3.3. Instrumentation and Experimental Conditions

LC-MS/MS analysis was performed using a nanoAcquity UPLC system with a binary pump (Waters, Milford, MA, USA) interfaced with 6500 Q-TRAP (AB Sciex, Framingham, MA, USA) mass spectrometer. The Analyst software (Sciex, Framingham, MA, USA) was used for data acquisition. Glycopeptides were separated using a 150 mm × 75 μm HALO^®^ penta-HILIC column packed with 2.7 μm-diameter superficially porous particles (Advanced Materials Technology, Wilmington, DE, USA). The mobile phase consisted of 0.1% formic acid in water (A) and 0.1% formic acid in acetonitrile (B). The gradient program was optimized to enhance, as much as possible, the resolution of all glycopeptides analyzed in this study. The optimized gradient program [(min)/% B] was 0/85, 5/85, 50/60, 75/30, 85/30, 87/85, and 100/85. The mobile-phase flow rate was maintained at 400 nL/min. The injection volume was 1 μL, samples were thermostated at 15 °C, and column temperature was 40 °C. MS analysis was performed in the selected reaction monitoring (SRM) mode. The specific SRM transitions (see Table S1) were selected according to our previous work [14,20], in which the studied N-glycopeptides were identified manually by the information-dependent MS acquisition method. The precursor masses of triply or quadruply charged ions were paired with intensive oxonium fragment ion masses (*m*/*z* 204.1 corresponded to HexNAc and *m*/*z* 274.1 corresponded to sialic acid—H_2_O). This SRM method has been chosen as it exhibits high signal-to-noise ratio. A collision energy of 50 V and declustering potential of 80 V were selected to achieve the best signal-to-noise ratio. The dwell time was set at 20 ms. The ion source was set as follows: curtain gas, 20 psi; ion spray voltage, 2300 V; ion source gas, 11 psi; interface heater temperature, 150 °C; entrance potential, 10 V; and collision exit potential, 13.

### 3.4. Prediction Model Construction

To construct our prediction model, we first calculated retention time of each studied glycoform relative to A2G2 glycoform attached to the same peptide moiety. (Bi-antennary glycoforms are typically found in high yields in most plasma glycoproteins and A2G2 glycoform can be commonly detected even without the need of a prior enzymatic desialylation.) Next, the median RRT and standard deviation were calculated for each glycoform, and its retention time window was determined. All glycopeptides shown in Table 1 were used for the calculation. We expected the Gaussian distribution of the RRT values; therefore, the limits were within ± three standard deviations from the median, which accounted for 99.7% of the probability distribution for the variable (real retention time). We observed multiple chromatographic peaks in some SRM chromatograms (see Figure S1) that most likely corresponded to glycopeptides with different isobaric glycan isomers, such as those being fucosylated on the core or the outer arm, sialylated on different arms (the six antenna or the three antenna), or being α2,3- or α2,6-sialylated [17,19]. For simplicity, we did not calculate the retention time window for each isomer, but we included each of them into the calculation. Thus, the prediction of retention time windows includes all isomeric specimen.

## 4. Conclusions

Mass spectrometry is a key tool for structural characterization of intact glycopeptides. However, to achieve high-confidence identification, information-rich MS/MS spectra are needed. If such spectra are not available for all analyzed glycopeptides, the confidence of their identification can be improved by including orthogonal information, such as those derived from chromatographic parameters into the search engine algorithms. Here, we propose a simple, proof-of-concept model predicting relative retention windows for glycopeptides that may serve this purpose. To develop the model, we determined retention behavior of several glycoforms of six tryptic peptides of haptoglobin, hemopexin, and sex hormone-binding globulin in nanoHILIC. The relative spacing between the glycoforms attached to the same peptide backbone varies minimally for different peptides. Hence, the retention of individual peptide glycoforms was expressed as a retention time relative to the respective bi-antennary glycoform. Based on the variance of the RRTs of individual glycoforms, we derived an easy-to-use mathematical model, and we have used it to successfully predict the retention time windows of fetuin glycopeptides in HILIC. Although the model was tested only on a limited number of glycopeptides, we consider the concept of the RRTs functional and suitable for being incorporated into search algorithms and tools in order to improve their glycopeptide identification confidence.

## Figures and Tables

**Table 1:** The glycopeptides of sex hormone-binding globulin, haptoglobin, and hemopexin used to predict retention time windows. Symbols: 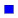, *N*-acetylglucosamine (GlcNAc); 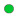, Mannose (Man); 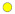, Galactose (Gal); 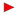, Fucose (Fuc); 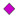, Sialic acid.

Sex-Hormone Binding Globulin					
SHEIWTHSCPQSPG**N**GTDASH LDVDQAL**N**R	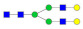 A2G2	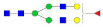 A2G2F1	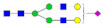 A2G2S1	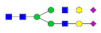 A2G2S2	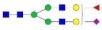 A2G2S1F1
	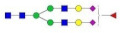 A2G2S2F1	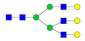 A3G3	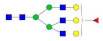 A3G3F1	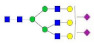 A3G3S2	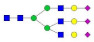 A3G3S3
Haptoglobin					
VVLHP**N**YSQVDIGLIK	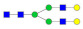 A2G2	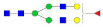 A2G2F1	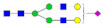 A2G2S1	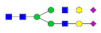 A2G2S2	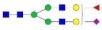 A2G2S1F1
	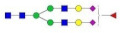 A2G2S2F1	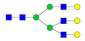 A3G3	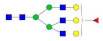 A3G3F1	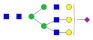 A3G3S1	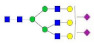 A3G3S2
	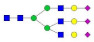 A3G3S3				
MVSHH**N**LTTGATLINEQWLLTTAK	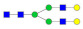 A2G2	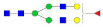 A2G2F1	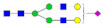 A2G2S1	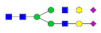 A2G2S2	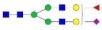 A2G2S1F1
	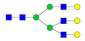 A3G3	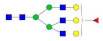 A3G3F1	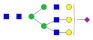 A3G3S1	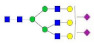 A3G3S2	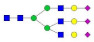 A3G3S3
Hemopexin					
SWPAVG**N**CSSALR	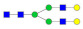 A2G2	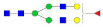 A2G2F1	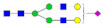 A2G2S1	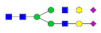 A2G2S2	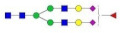 A2G2S2F1
	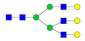 A3G3	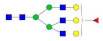 A3G3F1	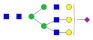 A3G3S1	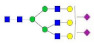 A3G3S2	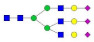 A3G3S3
ALPQPQ**N**VTSLLGCTH	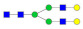 A2G2	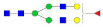 A2G2F1	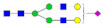 A2G2S1	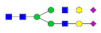 A2G2S2	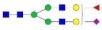 A2G2S1F1
	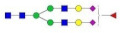 A2G2S2F1	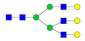 A3G3	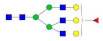 A3G3F1		

**Table 2:** Parameters used for calculation of retention time windows for different glycoforms.

	**A2G2F1**	**A3G3**	**A3G3F1**	**A2G2S1**	**A2G2S2**
Median	1.032	1.088	1.121	1.387	1.879
Lower limit	1.027	1.074	1.102	1.347	1.846
Upper limit	1.034	1.101	1.140	1.427	1.912
	**A2G2S1F1**	**A2G2S2F1**	**A3G3S1**	**A3G3S2**	**A3G3S3**
Median	1.410	1.892	1.447	1.953	2.307
Lower limit	1.388	1.813	1.372	1.886	2.232
Upper limit	1.433	1.971	1.522	2.019	2.381

Lower limit = median − 3σ; Upper limit = median + 3σ; σ is the standard deviation.

**Table 3:** Comparison of retention time windows predicted for fetuin glycopeptides with their actual measured retention times.

Identified Glycoforms	Predicted Retention Window (min)	Measured Retention Time (min)
LCPDCPLLAPL**N**DSR		
A2G2		30.2
A2G2S2	54.8–59.6	55.8
A3G3	32.5–33.3	32.9
A3G3S2	57.0–61.0	59.4
A3G3S3	67.5–71.9	68.1
RPTGEVYDIEIDTLETTCHVLDPTPLA**N**CSVR		
A2G2		36.5
A2G2S1	49.2–52.1	49.8
A2G2S2	66.2–72.0	67.1
A3G3	39.2–40.2	39.6
A3G3S1	50.7–52.3	52.1
A3G3S3	81.5–86.8	84.1
VVHAVEVALATFNAES**N**GSYLQLVEISR		
A2G2		33.1
A3G3	35.6–36.5	35.8
A3G3S3	73.9–78.7	75.7

## Supplementary Materials

The following supporting information can be downloaded at: https://www.mdpi.com/article/10.3390/molecules27123723/s1. Table S1: SRM transitions of studied glycopeptides. Figure S1: SRM chromatograms of hemopexin glycopeptides with their relative retention times [17]. Figure S2: SRM chromatograms of haptoglobin glycopeptides with their relative retention times. Figure S3: SRM chromatograms of sex-hormone binding globulin glycopeptides with their relative retention times.
