# Supplementary material for: Prediction of Intact N-Glycopeptide Retention Time Windows in Hydrophilic Interaction Liquid Chromatography
Source: Molecules. 2022 Jun 9;27(12):3723. doi: 10.3390/molecules27123723 (PMC9228347; doi:10.3390/molecules27123723)
Supplement: Supplementary file 1 [file molecules-27-03723-s001.zip › molecules-1740550-supplementary.pdf]

Supplementary Materials

Prediction of Intact N-glycopeptide Retention Time Windows in Hydrophilic Interaction Liquid Chromatography

Petr Kozlik [1,*], Katarina Molnarova [1], Tomas Jecmen [2], Tomas Krizek [1] and Zuzana Bosakova [1]

[1] Department of Analytical Chemistry, Faculty of Science, Charles University, Hlavova 8, 128 43 Prague 2, Czech Republic; katarina.molnarova@natur.cuni.cz (K.M.); krizek@natur.cuni.cz (T.K.); bosakova@natur.cuni.cz (Z.B.)

[2] Department of Biochemistry, Faculty of Science, Charles University, Hlavova 8, 128 43 Prague 2, Czech Republic; tomas.jecmen@natur.cuni.cz (T.J.)

***** Correspondence: kozlik@natur.cuni.cz (P.K.)

* Correspondence

RNDr. Petr Kozlík, Ph.D.

Charles University

Hlavova 8

Prague 2 -128 43

Czech Republic

Email: kozlik@natur.cuni.cz

Tel: 221 951 216

Table S1 SRM transitions of studied glycopeptides.

Glycopeptide	Precursor Ion (Da)	Product Ion (Da)
**Sex-hormone binding globulin**		
SHEIWTHSCPQSPG**N**GTDASH		
A2G2	982.6	204.1
A2G2F1	1019.2	204.1
A2G2S1	1055.4	204.1; 274.1
A2G2S2	1128.2	204.1; 274.1
A2G2S1F1	1091.9	204.1; 274.1
A2G2S2F1	1164.7	204.1; 274.1
A3G3	1073.9	204.1
A3G3F1	1110.4	204.1
A3G3S2	1128.2	204.1; 274.1
A3G3S3	1034.1	204.1; 274.1
LDVDQAL**N**R		
A2G2	889.4	204.1
A2G2F1	938.1	204.1
A2G2S1	986.4	204.1; 274.1
A2G2S2	1083.4	204.1; 274.1
A2G2S1F1	1035.1	204.1; 274.1
A2G2S2F1	1132.1	204.1; 274.1
A3G3	1011.1	204.1
A3G3F1	1059.8	204.1
A3G3S2	904.1	204.1; 274.1
A3G3S3	976.9	204.1; 274.1
**Haptoglobin**		
VVLHP**N**YSQVDIGLIK		
A2G2	1139.9	204.1
A2G2F1	891.7	204.1
A2G2S1	1237.0	204.1; 274.1
A2G2S2	1334.0	204.1; 274.1
A2G2S1F1	1285.6	204.1; 274.1
A2G2S2F1	1382.7	204.1; 274.1
A3G3	946.4	204.1
A3G3F1	982.9	204.1
A3G3S1	1358.7	204.1; 274.1
A3G3S2	1455.7	204.1; 274.1
A3G3S3	1552.7	204.1; 274.1
MVSHH**N**LTTGATLINEQWLLTTAK		
A2G2	1076.3	204.1
A2G2F1	1112.8	204.1
A2G2S1	1149.1	204.1; 274.1
A2G2S2	1221.9	204.1; 274.1
A2G2S1F1	1185.6	204.1; 274.1
A3G3	1167.5	204.1
A3G3F1	1204.0	204.1
A3G3S1	1240.4	204.1; 274.1
A3G3S2	1313.1	204.1; 274.1
A3G3S3	1385.9	204.1; 274.1
**Hemopexin**		
SWPAVG**N**CSSALR		
A2G2	1009.7	204.1
A2G2F1	1058.4	204.1
A2G2S1	1106.8	204.1; 274.1
A2G2S2	1203.8	204.1; 274.1
A2G2S2F1	939.6	204.1; 274.1
A3G3	1131.4	204.1
A3G3F1	1180.1	204.1
A3G3S1	1228.5	204.1; 274.1
A3G3S2	994.4	204.1; 274.1

A3G3S3	1067.2	204.1; 274.1
ALPQPQ**N**VTSLLGCTH		
A2G2	1120.2	204.1
A2G2F1	1168.8	204.1
A2G2S1	913.1	204.1; 274.1
A2G2S2	985.9	204.1; 274.1
A2G2S1F1	949.7	204.1; 274.1
A2G2S2F1	1022.4	204.1; 274.1
A3G3	1241.8	204.1
A3G3F1	968.2	204.1
**Fetuin**		
LCPDCPLLAPL**N**DSR		
A2G2	841.6	204.1
A2G2S2	987.4	204.1; 274.1
A3G3	932.9	204.1
A3G3S2	1078.6	204.1; 274.1
A3G3S3	1151.4	204.1; 274.1
RPTGEVYDIEIDTLETTCHVLDPTPLA**N**CSVR		
A2G2	1283.9	204.1
A2G2S1	1397.2	204.1; 274.1
A2G2S2	1469.9	204.1; 274.1
A3G3	1415.7	204.1
A3G3S1	1488.4	204.1; 274.1
A3G3S3	1633.9	204.1; 274.1
VVHAVEVALATFNAES**N**GSYLQLVEISR		
A2G2	1120.1	204.1
A3G3	1261.9	204.1
A3G3S3	1470.2	204.1; 274.1

SWPAVGNCSSALR

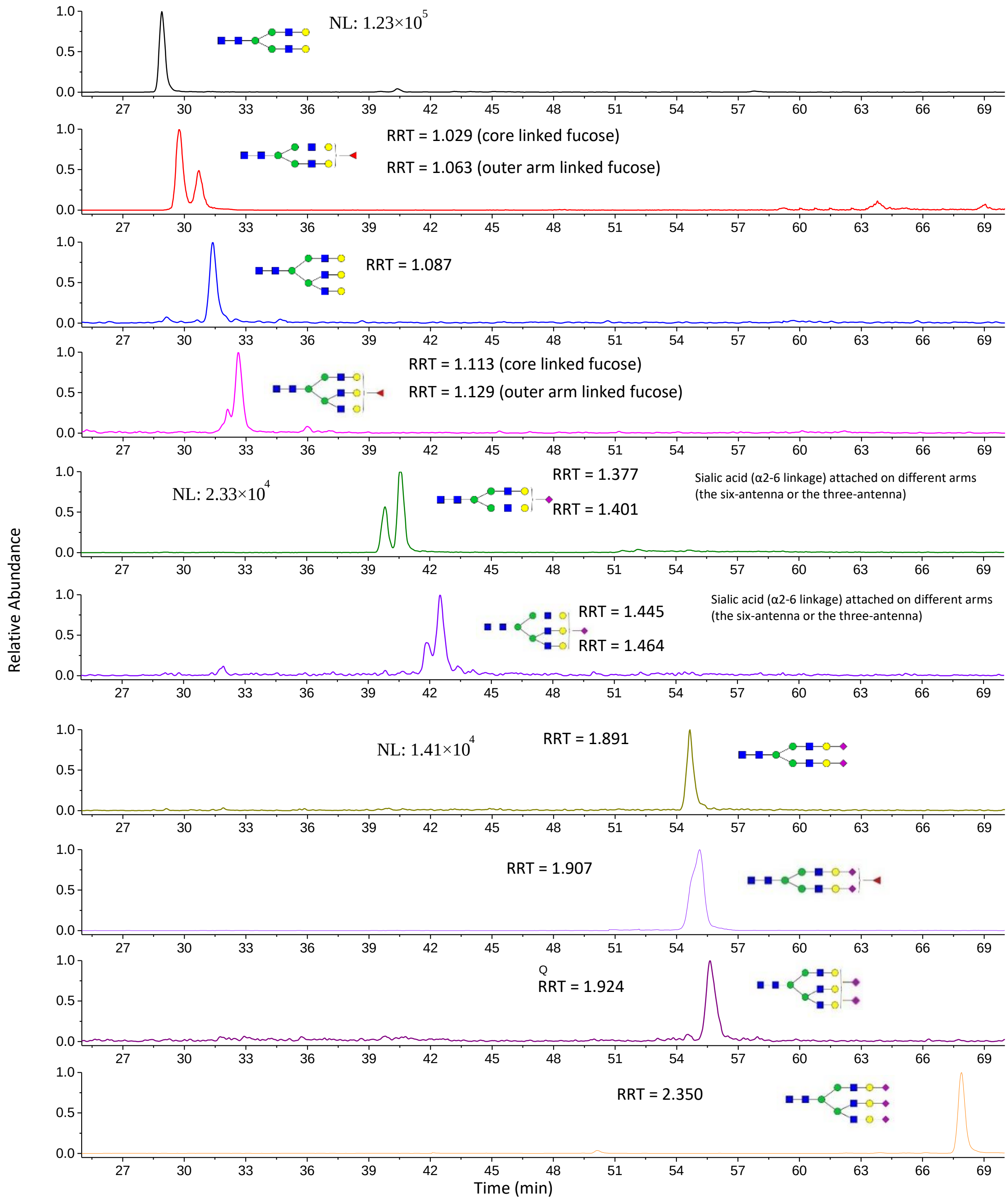

ALPQPQ**N**VTSLLGCTH

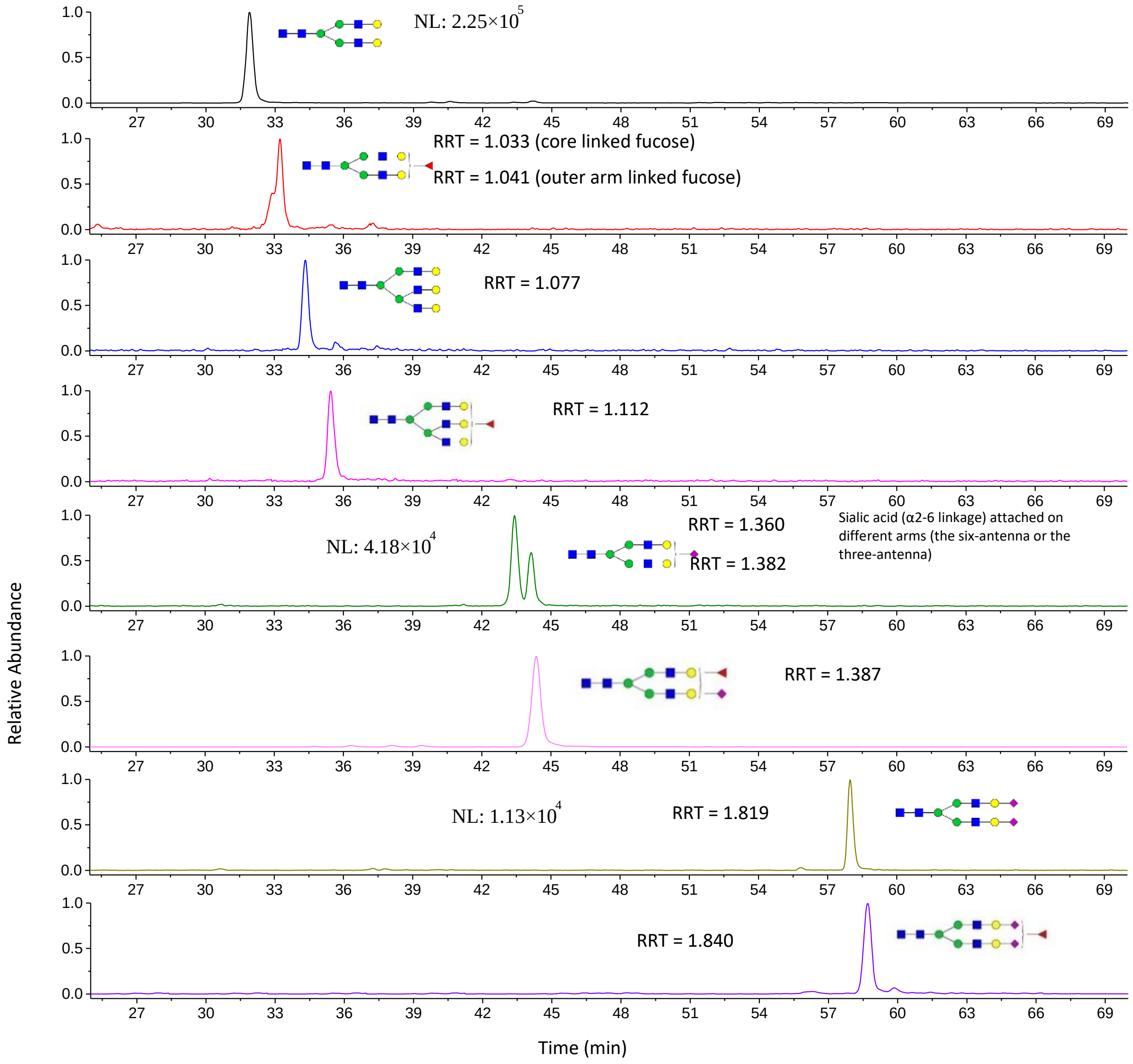

Figure S1 SRM chromatograms of hemopexin glycopeptides with their relative retention times. Characterization of glycopeptide isomers by exoglycosidase digestion was shown in paper [Kozlik, P.; Goldman, R.; Sanda, M. Hydrophilic interaction liquid chromatography in the separation of glycopeptides and their isomers. Anal. Bioanal. Chem. 2018, 410, 5001-5008, doi:10.1007/s00216-018-1150-3.].

MVSHHNLTTGATLINEQWLLTTAK

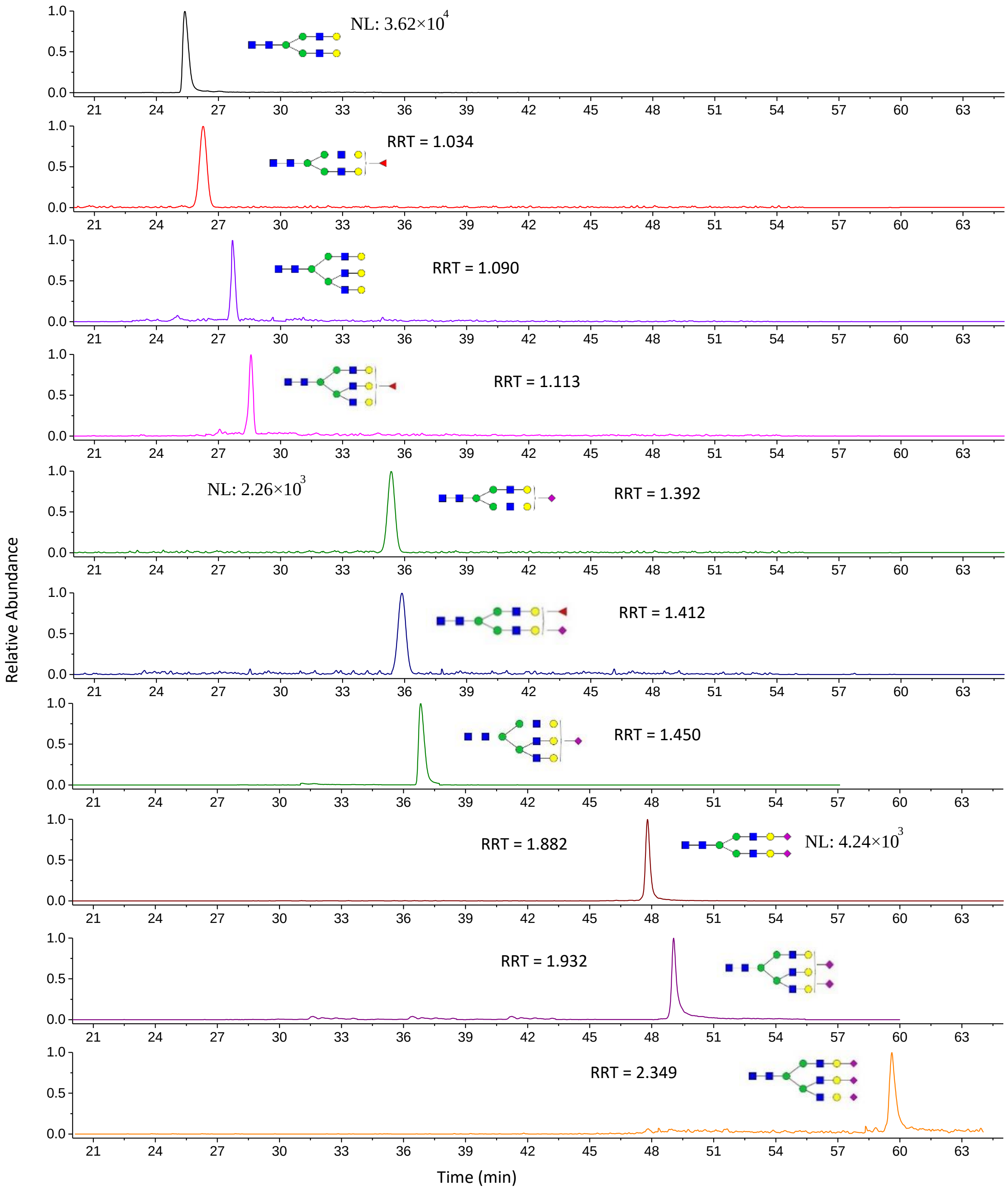

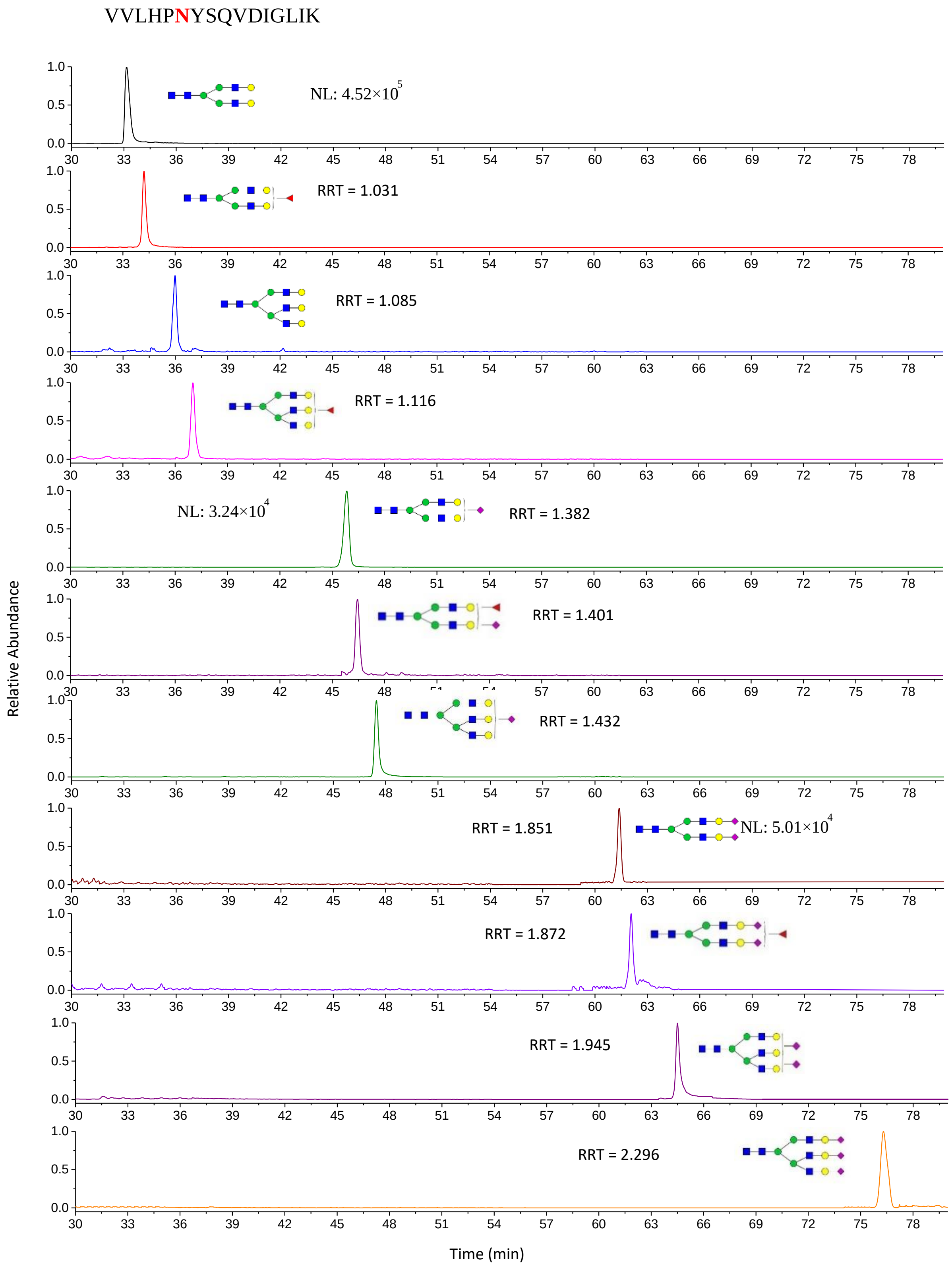

Figure S2 SRM chromatograms of haptoglobin glycopeptides with their relative retention times.

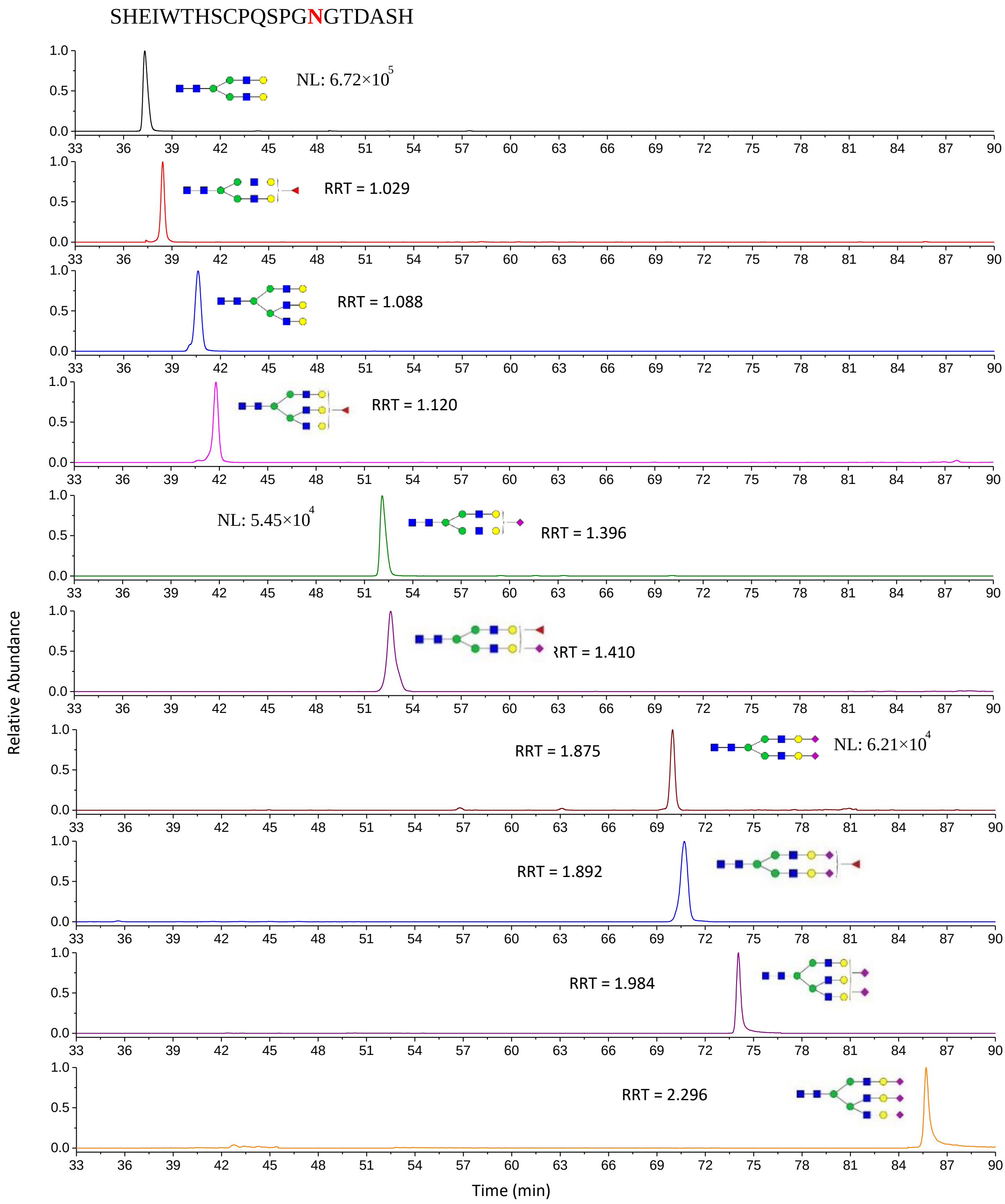

SHEIWTHSCPQSPGNGTDASH
NL: 6.72×10^5
RRT = 1.029
RRT = 1.088
RRT = 1.120
NL: 5.45×10^4
RRT = 1.396
RRT = 1.410
RRT = 1.875
NL: 6.21×10^4
RRT = 1.892
RRT = 1.984
RRT = 2.296
Relative Abundance
Time (min)

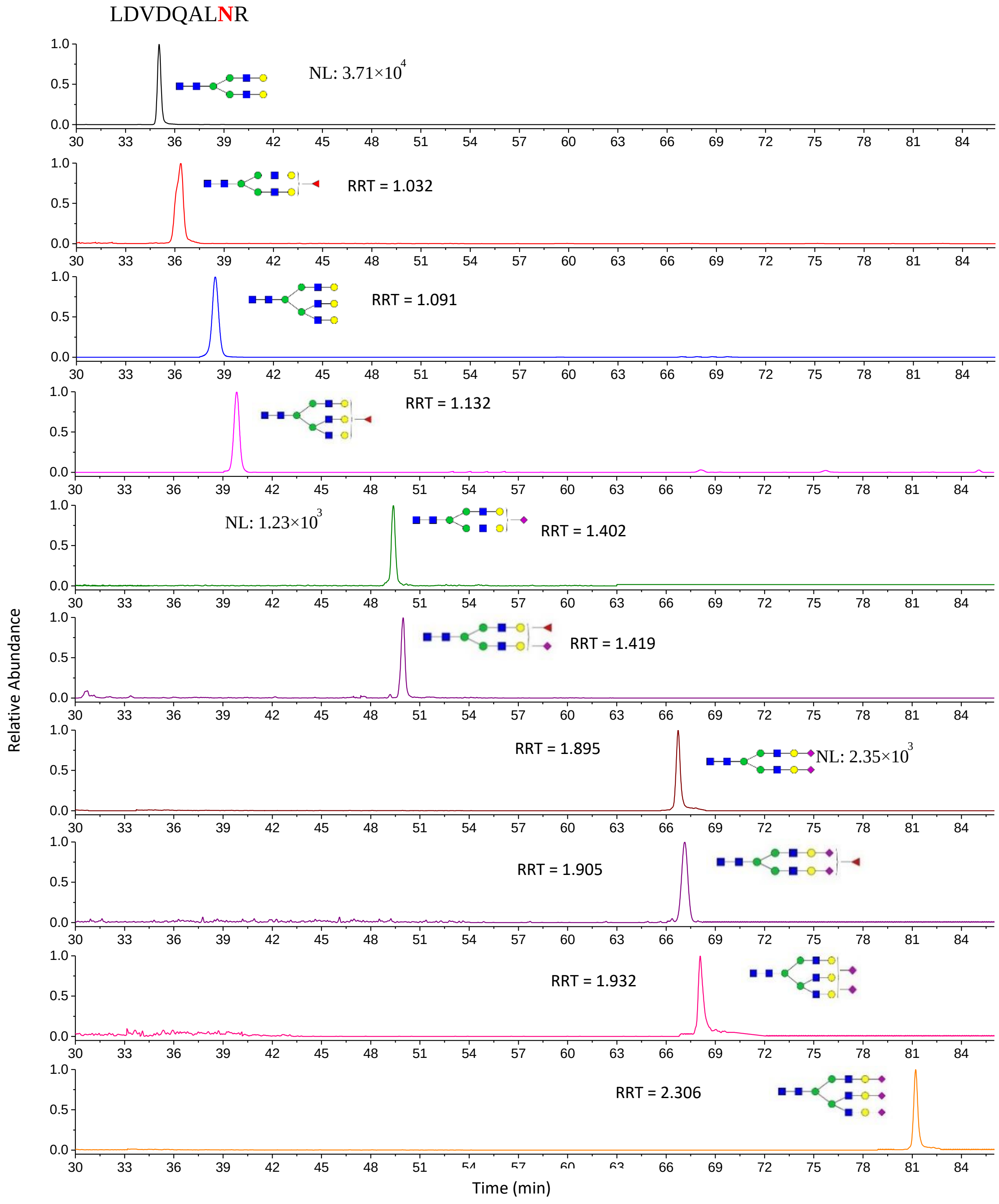

Figure S3 SRM chromatograms of sex-hormone binding globulin glycopeptides with their relative retention times.
